# Evaluation of Blended Learning Method Versus Traditional Learning Method of Clinical Examination Skills in Physiology Among Undergraduate Medical Students in an Indian Medical College

**DOI:** 10.7759/cureus.37886

**Published:** 2023-04-20

**Authors:** Ayesha Juhi, Mohammed Jaffer Pinjar, Gujaram Marndi, Bhagyashri R Hungund, Himel Mondal

**Affiliations:** 1 Physiology, All India Institute of Medical Sciences, Deoghar, Deoghar, IND; 2 Pharmacology, Dharanidhar Medical College and Hospital, Keonjhar, IND; 3 Pathology, Jawaharlal Nehru Medical College, Belgavi, IND

**Keywords:** mbbs student, e-learning, medical students, physicians, learning, clinical competence, online teaching, blended learning, traditional learning, clinical examination skills

## Abstract

Introduction

Clinical skills are crucial for medical professionals and are a vital part of a physician's identity. Medical students start learning these skills during their pre-clinical years of study. However, little research has been done on how novice medical students learn to improve these skills. Along with traditional teaching-learning methods, an approach to incorporating e-learning into medical education is through blended learning, which combines traditional classroom instruction with online learning activities.

Objective

This study aimed to compare the effectiveness of blended learning and traditional learning methods in teaching clinical examination skills to first-year undergraduate medical students by evaluating the objective structured clinical examination (OSCE) test scores.

Methodology

This was a two-arm prospective cross-over randomized study involving first-year MBBS students. The experimental group (group A) received blended learning, while the control group (group B) received traditional learning for the cardiovascular system examination (phase 1). The groups were then switched for the respiratory system examination (phase 2). An unpaired student t-test was used to compare the mean OSCE scores between the experimental and control groups in each phase, with statistical significance defined as a p-value < 0.05.

Results

The study involved 25 students in each group during phase 1 and 22 students in each group during phase 2. The experimental group had a mean age of 18.4 (±0.96) years in phase 1 and 18.35 (±1) years in phase 2, while the control group had a mean age of 18.06 (±1.04) years in phase 1 and 18.55 (±0.74) years in phase 2. In phase 1, the experimental group had a higher mean OSCE score (43 {±2.92}) than the control group (26.4 {±2}) (p <0.001). After switching in phase 2, the experimental group (previously the control group) had a higher mean OSCE score (47.82 {±1.68}) than the control group (33.59 {±1.59}) (p <0.001).

Conclusion

Blended learning is more effective than traditional learning in teaching clinical examination skills to medical undergraduate students. This study suggests that blended learning has the potential to replace the traditional method of learning clinical skills.

## Introduction

Imparting effective medical education to future doctors has been a long-standing challenge. Clinical examination skills are a crucial part of medical diagnosis and a core competency under India's competency-based medical education (CBME) curriculum [1]. Medical students first learn these skills during their pre-clinical year of study in physiology. However, there has been little exploration of the most effective way to teach these skills to novice learners [2].

There is a paucity of research on the optimal method of imparting clinical examination skills to novice learners. The traditional approach typically involves in-person, face-to-face instruction, during which medical students are trained in clinical examination skills by practicing on healthy subjects or real patients under the supervision of experienced clinicians or medical educators. Practical sessions utilizing the Demonstration, Observation, Assistance, and Performance (DOAP) framework are commonly used to provide students with the opportunity to observe a demonstration, assist the person performing, practice in a simulated setting, receive guidance while performing, or perform on their own [3]. However, the limited availability of time often restricts these sessions to one or two instances, which is inadequate for students to practice effectively and hone their clinical examination skills according to their individual learning needs.

In recent years, technology has become an indispensable part of our daily lives, and medical education is no exception. With the advent of e-learning, medical educators are now able to develop innovative educational tools that offer a more interactive, self-paced, and accessible approach to learning. E-learning has revolutionized medical education by allowing learners to study at their own convenience and pace and has been shown to be an effective way of delivering educational content [4].

One of the most effective approaches to incorporating e-learning into medical education is through blended learning, which combines traditional classroom instruction with online learning activities. Blended learning can offer a more comprehensive and engaging approach to learning, as it incorporates various multimedia elements such as videos, animations, quizzes, and interactive activities. By utilizing this approach, medical students are better able to grasp complex concepts and acquire skills more effectively, such as performing a physical examination [5]. Moreover, blended learning can be tailored to meet the specific needs of individual learners, allowing them to focus on areas where they need more practice and support.

In light of the increasing popularity of blended learning in medical education, we aimed to investigate the effectiveness of this teaching method compared to traditional in-person instruction for acquiring clinical examination skills among first-year medical students in an Indian medical college. Our research question was whether blended learning could improve students' clinical examination skills more effectively than the traditional method. To answer this question, we conducted a study in which one group of students received a session of blended learning, while the other group received traditional in-person instruction. We evaluated the clinical examination scores of both groups and compared them to determine the effectiveness of each teaching method.

## Materials and methods

Type and setting

A prospective, randomized, two-arm crossover study was conducted with first-year MBBS students enrolled in the Department of Physiology at Apollo Institute of Medical Sciences, Hyderabad, India. The data collection spanned from October 2019 to February 2020.

Ethics

This study was conducted in accordance with the ethical guidelines of the Apollo Institute of Medical Sciences, Hyderabad, and was approved by the institute's Institutional Review Board with reference number AIMSR/IRB/RC/2019/12/082. The study was conducted as a project under the advanced course in medical education. Before the initiation of the study, written informed consent was obtained from all participating students.

Randomization

**Figure 1:**
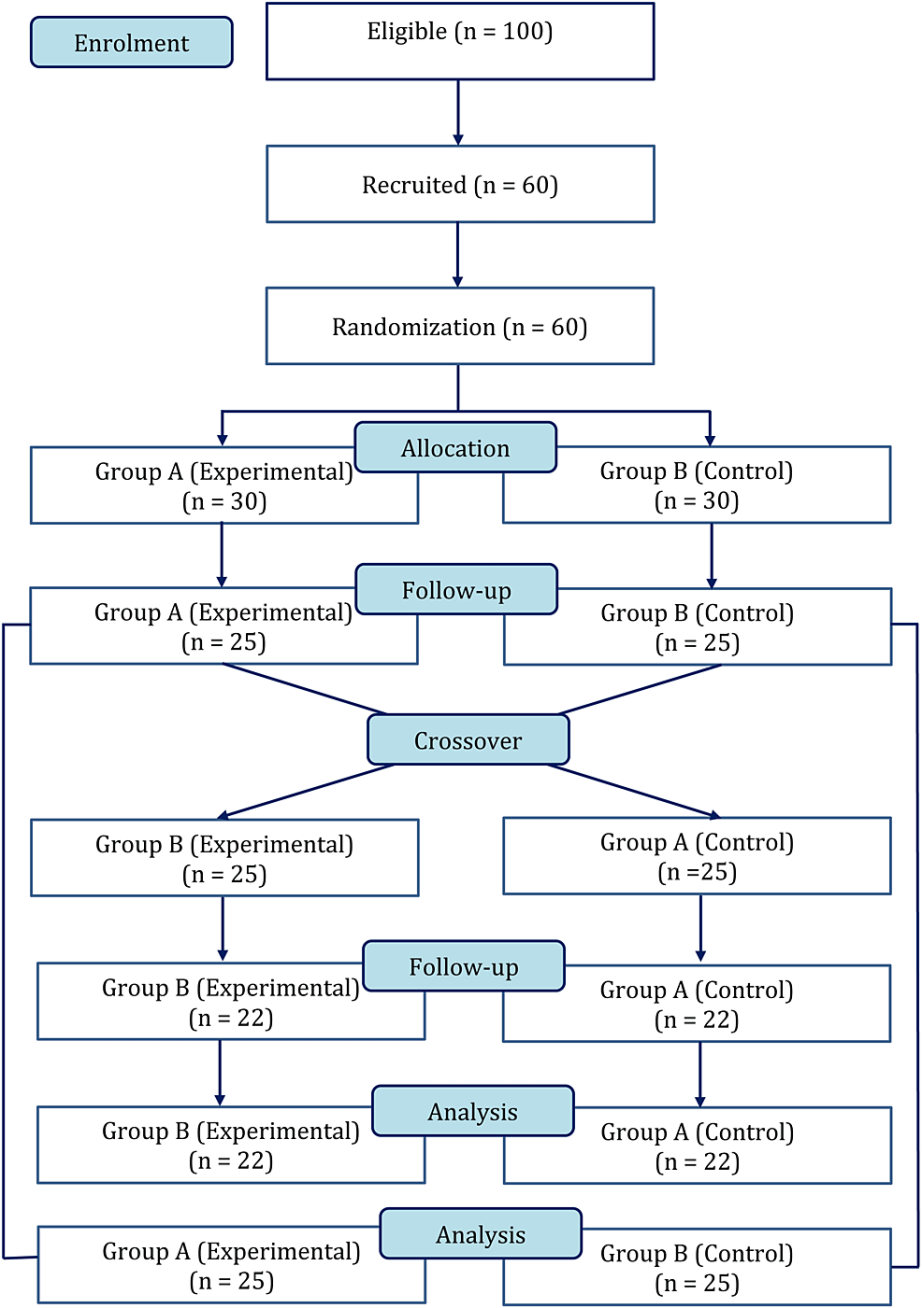
Sample size, recruitment, allocation, follow-up, and analysis in a modified consolidated standard of reporting trial flow chart

To conduct this study, we randomly selected 60 first-year MBBS students from a pool of 100 students at the Apollo Institute of Medical Sciences in Hyderabad. The students were then randomly assigned to either the experimental or control group, with 30 participants in each group. The entire process of recruitment, allocation, follow-up, and analysis is summarized in Figure 1, following the Consolidated Standards of Reporting Trials (CONSORT) guidelines.

The study was conducted in two phases, with Group A being in the experimental group and Group B being in the control group during phase 1, and then switching roles during phase 2, with Group B being the experimental group and Group A being the control group.

Phase 1 OSCE

**Figure 2:**
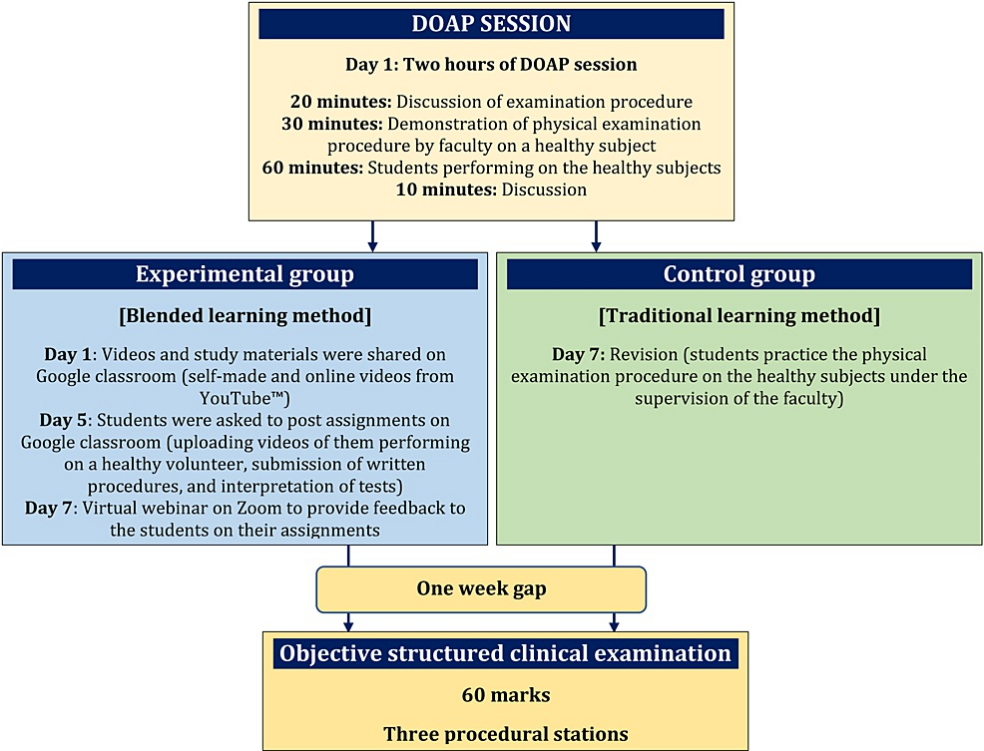
A brief of the method used for blended learning and traditional learning method DOAP: Demonstration, Observation, Assistance, and Performance

For the first phase of the study, we chose to evaluate the clinical examination of the cardiovascular system. We selected a single faculty member from the Department of Physiology to implement DOAP sessions for both the experimental and control groups. Another faculty member conducted an online session for the experimental group as part of the blended learning approach. Following the training, we conducted an examination using the objective structured clinical examination (OSCE) with three stations on three different topics of cardiovascular physical examination. Please refer to Figure 2 for a summary of the methods used.

Phase 2 OSCE

After a two-week gap, the groups were crossed over for phase 2 of the study, with group A becoming the control group and group B becoming the experimental group. The clinical examination of the respiratory system was chosen for phase 2, and the same process was followed as in phase 1, with a single faculty member from the Department of Physiology implementing DOAP sessions for both groups, and an online session for blended learning conducted for the experimental group by another faculty member. After completion of the training, an examination was conducted following the OSCE with a total of three OSCE stations having three topics of clinical examination of the respiratory system.

Preparation for the OSCE

A group of three faculty members from the Department of Physiology, General Medicine, and Pediatrics collaborated to prepare the OSCE answer checklist. Initially, each member individually created a checklist and later came to a consensus to produce the final version. All the OSCE checklists were prepared beforehand and stored in the question bank after checking the optimum validity and reliability of the questions. We took the OSCE for this study from that question bank. A sample of the OSCE is available in the appendices.

Data collection

The students underwent the examination, and a single rater rated them on the OSCE checklist (available as Annexure 1 for phase 1 and Annexure 2 for phase 2) in a blinded manner, i.e., without knowledge of the allocated group of students. The final scores were recorded and stored for further analysis.

Statistical analysis

The data was first assessed for normal distribution using the Shapiro-Wilk test. To compare the means of OSCE scores, an unpaired Student's t-test was used. Statistical analysis was performed using GraphPad Prism 7 software (GraphPad Software, San Diego, CA). A p-value of less than 0.05 was considered statistically significant.

## Results

**Figure 3:**
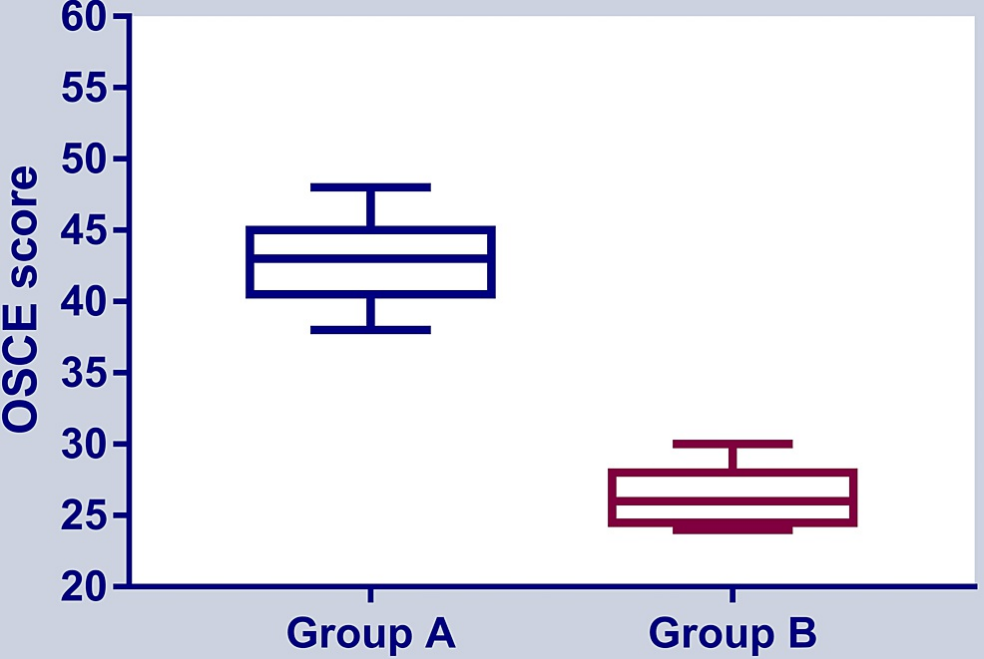
The OSCE score in phase 1 of the experiment OSCE: Objective structured clinical examination Group A was exposed to blended learning

We allocated 30 students to each group, but five students were dropped due to absence in one or more sessions. Therefore, in phase 1, 25 students with a mean age of 18.4 (±0.96) years comprised the experimental group, and 25 students with a mean age of 18.06 (±1.04) years comprised the control group. The mean OSCE score in the experimental group (43 {±2.92}) was significantly higher than the control group's score (26.4 {±2}) (p <0.001), as shown in Figure 3.

**Figure 4:**
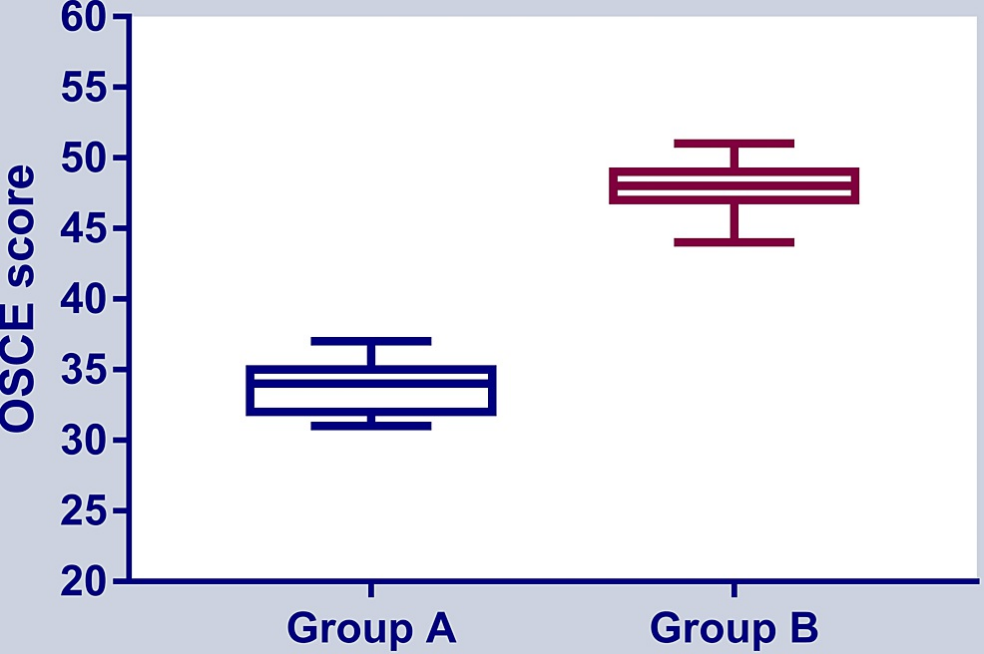
The OSCE score in phase 2 of the experiment OSCE: Objective structured clinical examination Group B was exposed to blended learning

In the second phase of the study, conducted 14 days after the first session, eight students were dropped, and the final sample was 22 participants. Their mean age was 18.35 (±1) years in the experimental group and 18.55 (±0.74) years in the control group. In this phase, the experimental group (previously the control group) also demonstrated a higher score in OSCE (47.82 {±1.68}) than the control group (33.59 {±1.59}) (p <0.001), as shown in Figure 4.

## Discussion

The study results revealed that using blended learning for teaching physical examination skills led to significantly higher scores in OSCE compared to traditional learning methods. Several factors may contribute to this finding, with one possible explanation being reinforced learning [6]. Blended learning combines online learning with traditional classroom instruction, providing learners with an enhanced learning experience. By integrating online tools and resources, blended learning can offer a more interactive and personalized approach to education, resulting in increased student engagement and motivation [7].

During blended learning, learners are exposed to video-assisted teaching through step-by-step procedure demonstrations. These videos can be accessed from any place and at any time, allowing learners to improve their skills at their own pace. Online discussion sessions provide opportunities for learners to clarify any doubts or rectify any errors in the procedure. Online quizzes, assignments, and assessments can be used to evaluate student learning and provide feedback [8]. These assessments can help students identify areas where they need to improve, and instructors can tailor their teaching to address these areas.

While blended learning offers many advantages, there are also some potential drawbacks to consider. One major concern is the need for access to technology and a reliable internet connection. In developing countries like India, some students may not have the necessary equipment or internet connectivity to participate in the online components of the course fully. Additionally, the online component of blended learning requires self-discipline and time-management skills [9]. Moreover, instructors need to invest significant time and effort in planning and preparing to ensure that the online and in-person components are well-integrated and complementary, which can be a challenge for some educators. Despite these limitations, many Indian medical colleges are gradually adopting online learning methods [10].

Enoch et al. found that students in a South African medical school performed better with blended learning than with only e-learning or face-to-face learning [11]. In the UK, students appreciated e-learning, but their engagement with such learning environments varied. Students perceived e-learning as being just as beneficial as other conventional methods of teaching clinical skills and recognized its incorporation in a blended approach [12]. According to Jang and Kim's study, OSCE videos have overall beneficial effects on students' acquisition of clinical skills. To help students use these materials more efficiently, faculty should include them in their lessons, add interactive tools to the e-learning environment to encourage participation, and use mobile devices for easy access [13]. Additionally, a review article by Coyne et al. suggested that blended learning not only enhances the knowledge and skills of students but is also frequently favored by them because of its adaptable nature [14]. Our study findings from an Indian medical institute support these findings.

This study was conducted at a single center located in the southern part of India. Therefore, it is important to note that the sample used may not be representative of all medical students in India. Additionally, due to some participants being lost to follow-up, the sample size in each group was reduced. Furthermore, this study was limited to only first-year undergraduate students pursuing MBBS. To gain a more comprehensive understanding of the applicability of blended learning in medical education, further research should be conducted with students studying clinical subjects.

## Conclusions

Blended learning has been found to improve clinical examination skills among medical undergraduate students compared to traditional learning methods. This suggests that it has the potential to replace traditional methods of learning clinical skills. While the gold standard for learning clinical examination skills remains face-to-face physical examination of patients at the bedside, blended learning can reinforce these skills and help instill the fundamentals of clinical medicine in first-year students. By incorporating blended learning into medical education, we can better prepare the physicians of tomorrow to be the first point of contact for their patients.

## Figures and Tables

**Figure 5:**
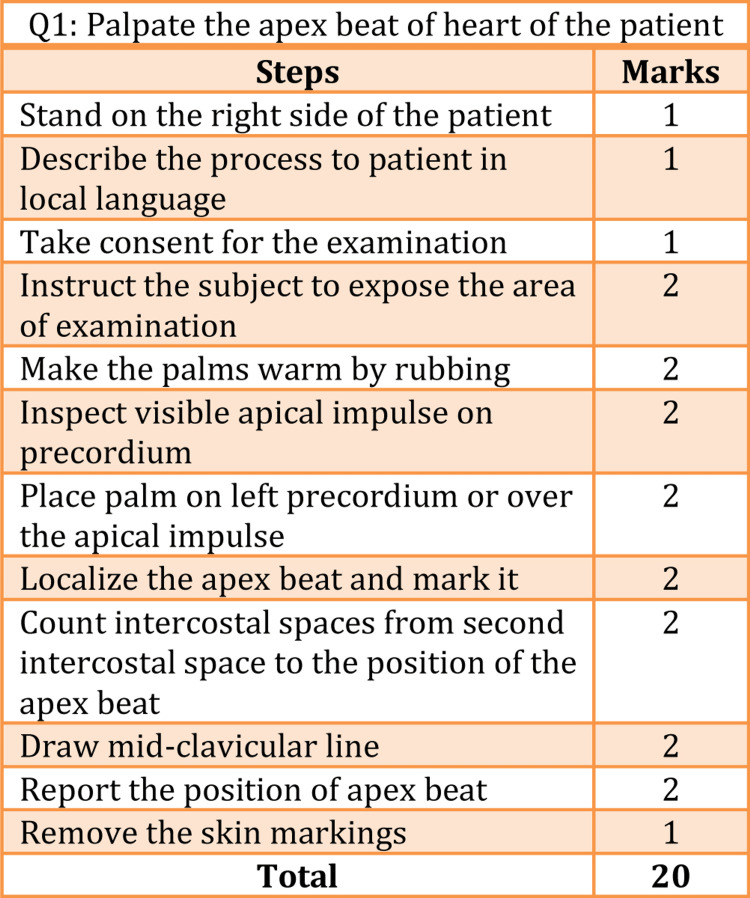
An example of an objective-structured clinical examination question (one of the three questions) in cardiovascular physiology

**Figure 6:**
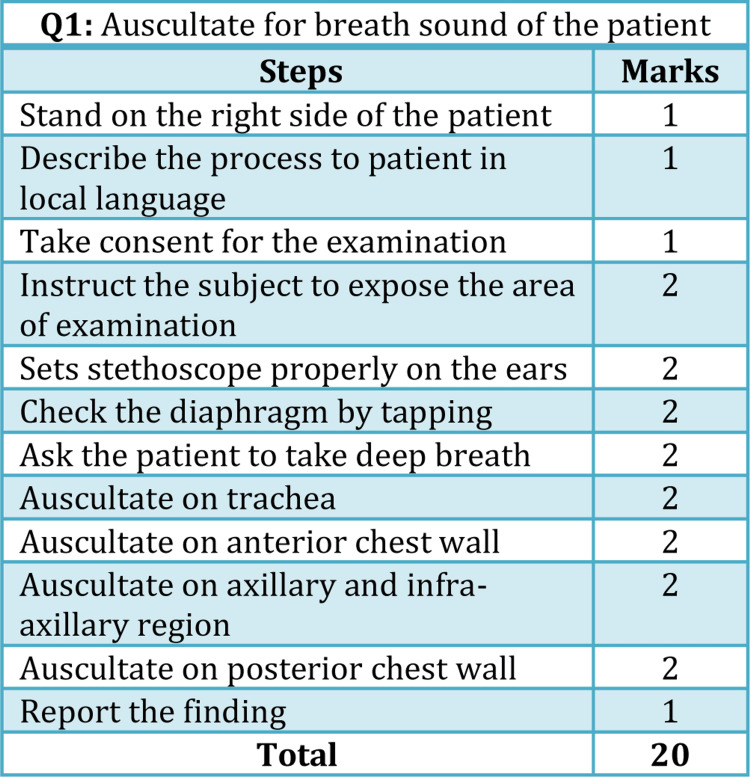
An example of an objective-structured clinical examination question (one of three questions) in respiratory physiology

## Appendices

Contribution details

Ayesha Juhi: Concept, methodology, data collection, interpretation of data, literature search, writing the manuscript, and approving the final version of the manuscript.

Mohammed Jaffer Pinjar: Design, literature search, editing manuscript, and approving the final version of the manuscript.

Gujaram Marndi: Interpretation of data, literature search, editing manuscript, and approving the final version of the manuscript.

Bhagyashri Hungund: Concept, supervision, editing manuscript, and approving the final version of the manuscript.

Himel Mondal: Data analysis, interpretation of results, visualization, literature search, editing manuscript, technical editing, and approving the final version of the manuscript.

Annexure 1

Annexure 2
